# Drug-Induced Acute Pancreatitis: An Evidence-Based Classification (Revised)

**DOI:** 10.14309/ctg.0000000000000621

**Published:** 2023-07-14

**Authors:** Jasmine Saini, Daniel Marino, Nison Badalov, Melanie Vugelman, Scott Tenner

**Affiliations:** 1 Maimonides Medical Center, State University of New York–Downstate Medical Center, Brooklyn, New York, USA.

**Keywords:** acute pancreatitis, drug-induced, medication-induced

## Abstract

**INTRODUCTION:**

Drug induced acute pancreatitis is a difficult diagnosis for clinicians. We previously published an “Evidence-Based Classification System” on Drug-Induced Acute Pancreatitis widely used by clinicians to assist in the identification of drugs. Unfortunately, this prior analysis based only on published case reports has been misunderstood. The prior review did not include studies with higher evidentiary value, such as randomized trials, case-control studies, and/or pharmacoepidemiologic studies. The use of the prior classification system has led to many patients being inappropriately labeled as having drug-induced acute pancreatitis. We now propose a “Revised” Evidence- Based Classification System for the purpose of determining which drugs cause acute pancreatitis based on the Grading of Recommendations, Development, and Evaluation criteria.

**METHODS:**

A search of the English Language literature was performed to identify all case reports with medication and/or drug induced acute pancreatitis. We divided the drugs implicated as causing acute pancreatitis into four groups based on the quality of evidence as defined by GRADE quality parameters.

**RESULTS:**

Although 141 drugs were identified in the literature as causing acute pancreatitis, only 106 drugs published in the literature as causing acute pancreatitis were high quality case reports. Only 3 drugs had evidence as causing acute pancreatitis from randomized controlled clinical trials, including 6-mercaptopurine and azathioprine.

**DISCUSSION:**

The vast majority of drugs implicated as causing acute pancreatitis in the literature have low or very low quality of evidence supporting those claims.

## INTRODUCTION

Acute pancreatitis is the most common gastrointestinal disease leading to hospitalization in the United States (1). A careful history, laboratory testing, and imaging reveals the etiology in most patients. The purpose of identifying the etiology is to prevent recurrent disease, including the possibility of more severe disease (2). However, in as many as a third of patients with acute pancreatitis, the etiology remains elusive. Idiopathic acute pancreatitis is defined as pancreatitis with no etiology established after initial laboratory (including triglyceride level) and imaging (transabdominal ultrasound, MRI, and/or computed tomography scan) tests (3). There have been hundreds of claims of possible causes of acute pancreatitis in these patients, including almost 200 medications (4). The claims of causation are based almost exclusively as case reports or case series with limited evidentiary value.

We previously published an “Evidence-Based Classification System” on Drug-Induced Acute Pancreatitis widely used by clinicians (5). Unfortunately, this prior analysis based only on published case reports has been misunderstood and often wrongly applied (6). For that analysis, we did not include studies with higher evidentiary value, such as randomized trials, case-control studies, and/or pharmacoepidemiologic studies. Despite the extensive list of drugs included in the prior classification, the classes were defined ONLY by the quantity and quality of published case reports and the demonstration of rechallenges and/or consistent latency among the case reports.

The general scientific method is the methodology that must be used in making causal claims about human health and disease (7). Epidemiological methods involving human subjects (study participants) are the most important means for identifying and testing hypotheses involving human disease causation. According to the United States Preventative Services Task Force, these studies are prioritized from strongest to weakest: randomized controlled trials (RCTs), controlled trials without randomization, cohort studies, case-control studies, case reports, and case series (8). Published case reports and case series are plagued by reporting bias and are considered the lowest quality of evidence.

As the evidentiary value of case reports to clinicians and applicability to patients with unexplained acute pancreatitis is profoundly limited, the importance of an evidence-based approach including an extensive review of RCT, cohort studies, and pharmacoepidemiologic analyses was needed. On the basis of this review, we now propose a “Revised Evidence-Based Classification System” for the purpose of determining which drugs cause acute pancreatitis based on the Grading of Recommendations, Development, and Evaluation criteria (9).

## METHODS

**Figure 1.**
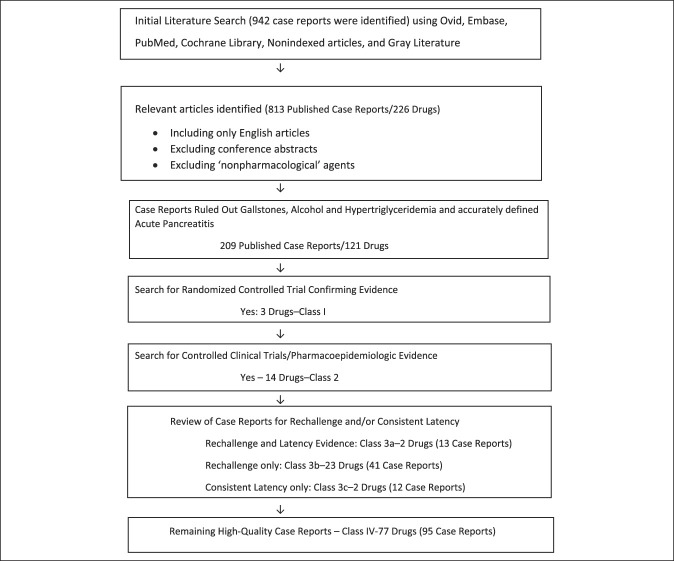
Method of evaluation of case reports and published studies.

A Medical Literature Analysis and Retrieval System Online search of the English language literature was performed to identify all case reports with medication and/or drug-induced acute pancreatitis using Medical Search Headings terms: drug induced, medication induced, drug associated, medication associated, and chemical induced with acute pancreatitis. Due to biased overreporting, pharmaceutical data, including the Physician Desk Reference and the US Food and Drug Administration (FDA) Adverse Reporting System, were not used in this analysis. Any drug or medication identified in case reports as having an association with acute pancreatitis led to a search of randomized controlled clinical trials and case-control trials, which listed adverse events, effects, side effects of the drug compared with those of placebo and/or other studied drugs to determine whether acute pancreatitis was increasingly associated with the studied drug (Figure 1). To be included, the diagnosis of acute pancreatitis needed to be established by The American College of Gastroenterology Guidelines: 2 of 3 criteria: (i) abdominal pain consistent with the disease, (ii) serum amylase and/or lipase greater than 3 times normal, and/or (iii) imaging (computed tomography or MRI) consistent with the diagnosis of acute pancreatitis (10).

The case reports were then separated into those case reports that were of high quality and those deemed not worthy of consideration. High-quality case reports were those that ruled out the more common causes of acute pancreatitis: gallstones, alcohol, and hypertriglyceridemia (see Supplementary Digital Content, http://links.lww.com/CTG/A981).

If ultrasound and/or MRI were not performed or gallstones were not described as being ruled out, the case report was deemed not worthy of consideration. A comment or laboratory parameter of the triglyceride level must have been reported for the case report to be included. In addition, a comment to the absence of alcohol abuse must have been included.

We divided the drugs implicated as causing acute pancreatitis into 4 groups based on the quality of evidence as defined by Grading of Recommendations, Development, and Evaluation quality parameters (Table 1). RCTs have the most evidence (class 1), while case-control and pharmacoepidemiologic studies come in a close second in evidentiary value (class 2). High-quality case reports were then divided into 2 classes. The first class (class 3) included high-quality case reports that had a rechallenge and/or a consistent latency. Case reports that had a rechallenge, where a drug is stopped and then restarted with another attack of acute pancreatitis, were considered better evidence. In addition, those drugs with 3 or more case reports with a consistent latency were also considered higher quality of evidence. If there were more than 3 case reports, three-fourths of the case reports need to have a consistent latency to be included. Consistent latency was defined as per our previous classification system. Latencies were defined as early (less than 24 hours), intermediate (1–30 days), and long (more than 30 days). A class 3a drug had both case reports with a rechallenge and a consistent latency. Class 3b and class 3c were case reports that had either a rechallenge or consistent latency. Last, if a drug had high-quality case reports but no case report had a rechallenge nor a consistent latency, due to the limited evidentiary value of case reports, they were considered class 4. All other case reports were deemed low quality and not included in the analysis. The authors felt that any case report that did not rule out the more common causes of acute pancreatitis, alcohol, gallstones, and/or hypertriglyceridemia, was not worthy of inclusion.

**Table 1.** Evidence-based classification for drug-induced acute pancreatitis–revised

Class 1. High Quality of Evidence for causation of acute pancreatitis: Randomized Controlled Clinical Trials.
Class 2. Moderate quality of evidence for causation of acute pancreatitis: Case-control studies and/or pharmacoepidemiology studies.
Class 3. Low-quality evidence for causation of acute pancreatitis: High-quality case reports.
Class 3a: Case reports showing “rechallenge and consistent latency”
Class 3b: Case report showing rechallenge only
Class 3c Case report showing consistent latency only.
Class 4. Very low-quality evidence: high-quality case reports but no rechallenge nor consistent latency.

## RESULTS

In our initial review, 226 drugs were identified in the literature as causing acute pancreatitis (Figure 1). One hundred five drugs were eliminated simply as poor quality, as previously reported. In the final analysis, we identified only 209 published case reports and 121 drugs published in the literature as causing acute pancreatitis with high-quality case reports. Table 2 summarizes which drugs have the highest quality of evidence as causing acute pancreatitis based on our further analysis. Only 3 drugs had evidence as causing acute pancreatitis from randomized controlled clinical trials, including 6-mercaptopurine and azathioprine (class 1). Only 14 drugs were shown from case-control trials or pharmacoepidemiologic studies as causing acute pancreatitis (class 2). Of published case reports, only 104 drugs had well-written case reports that excluded alcohol, gallstones, and hypertriglyceridemia and had a rechallenge or consistent latency among the reports. The cases were divided into 4 groups. We found 2 drugs that had evidence of both a rechallenge and consistent latency (class 3a), 23 drugs that had only a rechallenge (class 3b), and only 2 drugs with a consistent latency and lack of rechallenge among the high-quality case reports (class 3c). The last group of 95 remaining high-quality case reports yielded 77 drugs, which were considered the lowest quality of evidence of causation, class 4.

**Table 2.** Evidence-based list of drugs that cause acute pancreatitis

Class 1. Didanosine (18–20), azathioprine (21,22), 6-mercaptopurine (23)
Class 2. Acetaminophen^a^ (24), ACE inhibitors (25,26), Typical Antipsychotics (not atypical) (27), Benzodiazepines^a^ (28), DPP4 inhibitors (29), GLP1 agonists (30,31), Immune Checkpoint Inhibitors (32,33), Codeine (34), Methimazole (35,36), Metronidazole (37,38), Peg/L Asparaginase (39,40), Protease Inhibitors (41), SSRIs (42–44), Valproic Acid (45)
Class 3.
Class 3a: 5-ASA (46–54) Sulindac (55–58)
Class 3b: Acetaminophen-Codeine (59), Bezafibrate (60), Bortezomib (61,62), Cannabis (63–67), Capecitabine (68,69), Carbimazole (70,71), Estrogen (72), Fenofibrate (73), Isoniazid (74–76), Methyldopa (77), Nelfinavir (78), Nitrofurantoin (79,80), Pravastatin (81–83), Paclitaxel (84,85), Procainamide (86), Pyritinol (87), Rosuvastatin (88,89), simvastatin (90), Sulfasalazine (91,92), Thalidomide (93), Tetracycline (94–96) Trimethoprim-Sulfamethoxazole (97,98), Vemurafenib (99)
Class 3c: Doxycycline (100–104), Interferon alpha 2b and Ribavirin (105–111)
Class 4. Adefovir (112), Amiodarone (113), Arginine (114,115), Atorvastatin (116), Axitinib (117), Betamethasone/Roxithromycin (118), Canagliflozin (119), Carbamazepine intoxication (120), Candesartan (121), Celecoxib (122), Clozapine (123–125), Clomipramine^b^ (126),Cyclosporin (127), Danazol (128), Dapsone (129), Dexamethasone (130,131), Docetaxel (132), Doxorubicin and ifosfamide (133), Diclofenac (134), Dimethyl Fumarate (135), Eluxadoline (136,137), Erythromycin (138,139), Erythromycin/Lovastatin (140), Etoposide and Lobaplatin (141), Fluvastatin (142), Flurbiprofen (143), Furosemide (144), Growth Hormone (145), Hydrochlorothiazide/Irbesartan (146), Ibuprofen (147,148),^b^ Indomethacin (149,150), Ifosfamide (151), Interferon alpha 2a (152), Irbesartan (153), Itraconazole (154), Ketoprofen (155), Lamotrigine (156), Lanreotide (157), Loperamide (158), Mefenamic acid (159). Metformin (160,161), Metolazone (162), Methandrostenolone (163), Methylprednisolone (164), Methylprednisone/prednisone^b^ (165), Micafungin (166), Mirtazapine (167,168), Mycophenolate mofetil (169), Naproxen (170,171), Nicotine gum (172), Ocrelizumab (173), Octreotide (174,175), Ofloxacin-ornidazole (176), Omeprazole (177), Olanzapine (178), Pantoprazole (179), Penicillin (180), Phenolphthalein (181), Pentamidine (182), Posaconazole (183), Propofol (184–188), Ranitidine (189), Regorafenib (190), Riluzole (191), Risperidone (192), Rivaroxaban (193), Salicylazosulfapyridine (194), Secnidazole (195), Simvastatin/Salicylate (196), Sorafenib (197–199), sunitinib (197), Sunitinib/Axitinib (200),Tadalafil (201), Theophylline overdose (202), Tigecycline (203,204), Vedolizumab (205), Vismodegib (206)

a At toxic doses.

b At high dose.

## DISCUSSION

While the World Health Organization database lists more than 500 drugs as causing acute pancreatitis, there is a paucity of evidence that most of these drugs is the etiology of acute pancreatitis (4). Similarly, the US FDA adverse event database for “Medwatch” reports lists hundreds of medications as “possibly” causing acute pancreatitis with minimal evidence. The US FDA and others have concluded that these case reports have limited scientific “causal value” (11). The limitations of these spontaneous reporting systems include subjective and imprecise reporting practices, overreporting biases, and poor quality.

Clinicians may choose to stop a medication on a particular patient due to individual suspicion; however, blaming the drug as causing acute pancreatitis merely due to a prior published case report or cases series must be recognized as of limited scientific value. Case reports are simply too biased to rely on for causation. The fundamental problem with causal inference is the primary reason why RCTs are considered the gold standard of scientific research in therapeutic and etiologic research. Randomized trials provide the best approximation to solving this problem by assuming that individuals who do not receive the medication are similar as possible to the individuals who do not receive the medication (control group).

Although we used randomized trials for the best evidence of a drug causing or not causing acute pancreatitis, there are limitations to this approach. The trials may not have been designed in a method to identify cases of acute pancreatitis between the studied and control groups. Thus, adverse reporting for acute pancreatitis by research staff may have been underreported. In addition, the number of persons enrolled in the trials may have been too small to detect differences in the number of patients who develop acute pancreatitis. While these limitations are real, we believe that randomized trials still provide the best evidence for causation. The RCT is considered the most rigorous and robust research method of determining whether a cause-effect relation exists.

While epidemiologic studies, such as prospective cohort studies and case-control studies, do not have randomly identified controls, the careful selection of controls allows for another solution to the fundamental problem of bias. Case reports about disease causation that lack controls are of questionable validity and reliability. Although all adverse events begin as case reports (even if not actually reported in the scientific literature), it is important to emphasize that case reports only provide clues to etiology (12). If a clinician uses these case reports as evidence of causation, they are likely to be led astray as toward the best scientific explanation of the available evidence (13). At best, case reports and case series generate (rather than test) causal hypotheses about any relationship between medications and adverse events (14).

Largely due to tradition in the literature and the volume of publications, we chose to include case reports and to separate them into 4 classes and subclasses (classes 3a, 3b, 3c and class 4). However, we included only those case reports that had sufficient quality as defined by the Atlanta criteria and having ruled out other more common causes of acute pancreatitis. As other authors had appreciated the increased quality of evidence in case reports that had a rechallenge and/or a consistent latency, we considered these high-quality case reports separately as a class within class 3 (4,5,15). While a consistent latency provides some evidence of an underlying common mechanism, no biologic mechanism for any medication causing acute pancreatitis has been established (16). Four decades ago, Mallory and Kern (1980) included “rechallenge” as higher quality of evidence than a simple case report. However, few of the published case reports with rechallenge occur after a sufficient period after the attack of acute pancreatitis. In addition, the dosage, timing, and outcome are often missing. Clinicians should be aware that acute pancreatitis will recur in a third of patients within a year and as many as 10% within the first month of an attack of acute pancreatitis (3). Which is more likely, the drug was the cause in the rechallenge, or the acute pancreatitis would have recurred regardless of the drug?

In pharmacovigilance, the strongest evidence of causation of a drug are results from analytical studies that select appropriate control populations and provide evidence revealing a relative risk sufficiently strong to withstand the impact of bias and confounding. The only medications that have this level of evidence as causing acute pancreatitis are 6-MP and its metabolite, azathioprine. Didanosine also has similar clinical trial evidence as causing acute pancreatitis. The evidence is reproducible in multiple studies, including multiple RCTs. The analogy between the strength of evidence and metabolic similarity between 6-MP and azathioprine provides further evidence for these 2 drugs. The lack of a dose-response relationship and the inability to explain a biologic (or psychosocial) mechanism does not limit the evidence that these drugs cause acute pancreatitis (17).

In conclusion, most drugs implicated as causing acute pancreatitis in the literature have low or very low quality of evidence supporting those claims. Most drugs have been implicated as causing acute pancreatitis based on low-quality case reports rather than high-quality randomized trials, cohort studies, or pharmacoepidemiologic studies. In patients with acute pancreatitis who are taking 6-MP, azathioprine, or didanosine, the medication should be stopped because there is sufficient evidence that these drugs cause acute pancreatitis. However, stopping all the other medications in patients with idiopathic pancreatitis is unwarranted based on the evidence in the literature. While clinicians may stop a medication based on the fear that the drug may be the cause in a particular patient, relying on case reports for causation is inappropriate. Further study may identify idiosyncratic reasons that a particular person is susceptible to drug-induced acute pancreatitis. However, there is little scientific evidence that most medications believed to cause acute pancreatitis are the true etiology.

## CONFLICTS OF INTEREST

**Guarantor of the article:** Scott Tenner, MD.

**Specific author contributions:** J.S. and S.T.: manuscript was written. M.V.: proof-reading, verification of references was performed. J.S. and N.B.: data collection and analysis of case reports were performed. D.M. and S.T.: data collection and analysis of randomized controlled trials and large pharmacoepidemiologic databases were performed.

**Financial support:** None to report.

**Potential competing interests:** None to report.

**Study Highlights**

**WHAT IS KNOWN**

✓ Drugs have been implicated as causing acute pancreatitis.
✓ The best quality of evidence that a drug causes acute pancreatitis is from randomized controlled clinical trials and pharmacoepidemiologic databases.
✓ Well written case reports with a rechallenge and/or consistent latency can provide some evidence of causation. However, this is the weakest evidence of a drug causing acute pancreatitis.

**WHAT IS NEW HERE**

✓ Despite claims that numerous drugs cause acute pancreatitis, the evidence that a particular drug causes acute pancreatitis is quite limited.
✓ A Novel Classification System is shown based on the GRADE Criteria and will help guide clinicians in considering the evidence that a particular drug causes acute pancreatitis.
